# Therapeutic Potential of 5-HT_2C_ Receptor Ligands

**DOI:** 10.1100/tsw.2010.180

**Published:** 2010-09-14

**Authors:** Nanna H. Jensen, Thomas I. Cremers, Florence Sotty

**Affiliations:** ^1^ Department of Neurophysiology, H. Lundbeck A/S, Copenhagen-Valby, Denmark; ^2^ Brains On-Line LLC, South San Francisco, CA, USA

**Keywords:** serotonin 5-HT_2C_ receptors, dopamine, schizophrenia, depression, anxiety, obesity

## Abstract

Serotonin 2C receptors are G protein-coupled receptors expressed by GABAergic, glutamatergic, and dopaminergic neurons. Anatomically, they are present in various brain regions, including cortical areas, hippocampus, ventral midbrain, striatum, nucleus accumbens, hypothalamus, and amygdala. A large body of evidence supports a critical role of serotonin 2C receptors in mediating the interaction between serotonergic and dopaminergic systems, which is at the basis of their proposed involvement in the regulation of mood, affective behavior, and memory. In addition, their expression in specific neuronal populations in the hypothalamus would be critical for their role in the regulation of feeding behavior. Modulation of these receptors has therefore been proposed to be of interest in the search for novel pharmacological strategies for the treatment of various pathological conditions, including schizophrenia and mood disorders, as well as obesity. More precisely, blockade of serotonin 2C receptors has been suggested to provide antidepressant and anxiolytic benefit, while stimulation of these receptors may offer therapeutic benefit for the treatment of psychotic symptoms in schizophrenia and obesity. In addition, modulation of serotonin 2C receptors may offer cognitive-enhancing potential, albeit still a matter of debate. In the present review, the most compelling evidence from the literature is presented and tentative hypotheses with respect to existing controversies are outlined.

### INTRODUCTION

Serotonin (5-hydroxytryptamine, 5-HT) is a major neurotransmitter involved in a large number of physiological processes, such as regulation of feeding and energy balance, vomiting, aggression, mood, sex, perception, temperature regulation, and pain. A dysregulation in serotonergic neurotransmission has been proposed to be involved in a variety of disorders, including schizophrenia, depression, and anxiety[1,2,3,4].

In the brain, the ascending serotonergic system originates in the raphe complex, including mainly the dorsal and median raphe nuclei[5,6]. Serotonergic neurons in the raphe nuclei send widespread projections throughout the brain, where 5-HT exerts its effects through a variety of 5-HT receptors. The

5-HT receptor family consists of seven subfamilies termed 5-HT$_1$ through 5-HT$_7$, of which all are G protein-coupled receptors, except for the ionotropic 5-HT$_3$ receptor subfamily[3]. Until now, at least 15 distinct receptor subtypes of the 5-HT receptor family have been identified[7], of which the 5-HT$_{1A}$, 5-HT$_{1B}$, and 5-HT$_{1D}$ are autoreceptors[1,3,8]. The 5-HT$_{1C}$ receptor classification is vacant due to reclassification of this receptor to the 5-HT$_2$ family, so that this is now termed the 5-HT$_{2C}$ receptor[9,10].

Several 5-HT$_{2C}$ receptor ligands have been developed (Table 1). In this review, we will discuss the therapeutic potential of 5-HT$_{2C}$ receptor modulation in the treatment of schizophrenia, major depression, anxiety, and feeding and energy balance–related disorders.

**TABLE 1**
**Pharmacology of 5-HT$_{2C}$ Receptors**

| Compound Name | Chemical Name | Actions | Stage | Ref. |
|---|---|---|---|---|
| Agomelatine (S-20098) | N-[2-(7-methoxynaphthalen-1-yl)ethyl] acetamide | 5-HT$_{2B/2C}$ antagonist/melatonin agonist | Launched (Servier) | [84] |
| BVT-933 (PRX-00933) | Undisclosed | 5-HT$_{2C}$ agonist | Clinical Phase II (Proximagen) | [119] |
| CP-809101 | 2-(3-Chlorobenzyloxy)-6-(piperazin-1-yl)pyrazine | 5-HT$_{2C}$ agonist | Preclinical (Pfizer) | [35] |
| Lorcaserin (APD-356) | (1R)-8-chloro-2,3,4,5-tetrahydro-1-methyl-1H-3-benzazepine | 5-HT$_{2C}$ agonist | Preregistration (Arena Pharmaceuticals) | [118] |
| mCPP | m-Chlorophenylpiperazine | Dual 5-HT$_{1B/2C}$ agonist | N/A | [120] |
| RO60-0175 | (S)-2-(6-chloro-5-fluoroindol-1-yl)-1-methylethylamine) | 5-HT$_{2C}$ agonist | Preclinical (Hoffmann-La Roche) | [40] |
| RO60-0491 | (S)-2-(4,4,7-trimethyl-1,4-dihydro-indeno[1,2-b]pyrrol-1-yl)-1-methylethylamine | 5-HT$_{2C}$ antagonist | Preclinical (Hoffmann-La Roche) | [40] |
| S-32006 | N-pyridin-3-yl-1,2-dihydro-3H-benzo[e]indole-3-carboxamide | 5-HT$_{2C}$ antagonist | Preclinical (Servier) | [82] |
| SB-206533 | 5-Methyl-1-(3-pyridil-carbamoyl)-1,2,3,5-tetrahydropyrrolo[2,3 -f]indole | 5-HT$_{2B}$ antagonist/5-HT$_{2C}$ inverse agonist | Preclinical (GlaxoSmithKline) | [121] |
| SB-242084 | 6-Chloro-5-methyl-1-[[2-[(2-methyl-3-pyridyl)oxy]-5-pyridyl]carbamoyl]indoline | 5-HT$_{2C}$ antagonist | Preclinical (GlaxoSmithKline) | [39] |
| SB-243213 | 5-Methyl-1-[[2-[(2-methyl-3-pyridyl)oxy]- 5-pyridyl]carbamoyl]-6-trifluoromethylindoline | 5-HT$_{2C}$ inverse agonist | Preclinical (GlaxoSmithKline) | [122] |
| Vabicaserin (SCA-136) | (9aR,12aS)-4,5,6,7,9,9a,10,11,12,12a-decahydrocyclopenta[c][1,4]diazepino[6,7,1-ij]quinoline | 5-HT$_{2C}$ agonist | Clinical Phase II (Pfizer) | [123] |
| WAY-161503 | (4aR)-8,9-dichloro-2,3,4,4a-tetrahydro-1H-pyrazino[1,2-a]quinoxalin-5(6H)-one | Dual 5-HT$_{2B/2C}$ agonist | Preclinical (Pfizer) | [113] |
| WAY-163909 | (7bR, 10aR)-1,2,3,4,8,9,10,10a-octahydro-7bH-cyclopenta-[b][1,4]diazepino[6,7,1hi] indole | 5-HT$_{2C}$ agonist | Preclinical (Pfizer) | [111] |

## THE 5-$HT_{2C}$ RECEPTOR SUBTYPE

### Expression Pattern

The expression of the 5-$HT_{2C}$ receptor appears to be limited to the central nervous system (CNS). Messenger RNAs for the 5-$HT_{2C}$ receptor subtype are present in the choroid plexus, frontal cortex, hippocampus, hypothalamic nuclei, ventral tegmental area (VTA), substantia nigra (SN) pars compacta, and pars reticulata, as well as in the terminal regions of the nigrostriatal and mesolimbic dopaminergic pathways, namely the striatum and the nucleus accumbens, respectively[9,11,12]. The majority of 5-$HT_{2C}$ receptors are located postsynaptically to serotonergic terminals, i.e., on GABAergic, glutamatergic, and dopaminergic (DA) neurons, where they act as somatodendritic heteroreceptors[10,11]. In addition, 5-$HT_{2C}$ receptors have been reported to be present in the raphe nuclei, where they were initially suggested to act as autoreceptors[10], but were later shown to be expressed by GABAergic interneurons[13]. In the SN, 5-$HT_{2C}$ mRNA is coexpressed with glutamic acid decarboxylase, but not with tyrosine hydroxylase mRNAs, indicating that 5-$HT_{2C}$ receptors are expressed by GABAergic, but not DA neurons[11,14,15,16]. In contrast, 5-$HT_{2C}$ receptors are expressed by both GABAergic and DA neurons in the VTA[17]. Furthermore, 5-$HT_{2C}$ receptors have been shown to be present on GABAergic projection neurons, i.e., medium spiny neurons in the nucleus accumbens and striatum[9]. Taken together, these anatomical findings suggest that 5-$HT_{2C}$ receptors are in a key position to modulate DA neurotransmission in the mesolimbic and nigrostriatal pathways.

### RNA Editing

The 5-$HT_{2C}$ receptor is the only known G protein-coupled receptor that undergoes RNA editing, leading to at least 14 functionally distinct isoforms with discrete basal receptor activity, desensitization rates, and drug-induced functional activation[3,18,19,20]. Interestingly, the existence of different 5-$HT_{2C}$ receptor isoforms resulting from RNA editing, together with their differential brain localization, opens new possibilities with respect to development of isoform-selective pharmacological agents with potentially improved therapeutic benefit and side effect profile[18]. In line with this, altered patterns of 5-$HT_{2C}$ RNA editing have been linked to several disorders, including schizophrenia and depression[20,21,22,23].

### Signaling Pathways

Intracellularly, 5-$HT_{2C}$ receptors are mainly coupled to $G_q$ proteins, thereby activating phospholipase C, leading to hydrolysis of phosphatidylinositol bisphosphate and the generation of the second messenger inositol triphosphate, ultimately resulting in an increase in intracellular calcium. However, it should be mentioned that the 5-$HT_{2C}$ receptor also couples to other pathways, such as phospholipase $A_2$ signaling pathway and phospholipase D via $G_{\alpha 13}$[11,17]. Interestingly, the signaling cascade associated with the receptor seems to vary with the different isoforms of 5-$HT_{2C}$ receptors resulting from RNA editing[18,19]. Moreover, activation of 5-$HT_{2C}$ receptors has been reported to induce a rapid desensitization[24].

## MODULATION OF DOPAMINERGIC NEUROTRANSMISSION IN THE MESOLIMBIC AND NIGROSTRIATAL PATHWAYS BY 5-$HT_{2C}$ RECEPTORS

Several studies have suggested that 5-$HT_{2C}$ receptors exhibit a modulatory action on the activity of DA neurons in the VTA and SN[25,26]. For instance, the nonselective 5-$HT_{2C}$ receptor agonist mCPP was shown to reduce both the basal firing rate and bursting activity of DA neurons in the VTA[26,27]. Further strengthening a role of 5-$HT_{2C}$ receptors in mCPP-mediated effects, the selective 5-$HT_{2C}$ receptor antagonist SB-242084 was shown to enhance DA levels in the nucleus accumbens, an effect attributed to the disinhibition of DA firing via 5-$HT_{2C}$ receptors expressed on GABAergic interneurons in the VTA[17]. In agreement with this, SB-242084 dose dependently increased the firing rate and bursting activity of DA neurons in the VTA[28]. Behaviorally, SB-242084 was found to potentiate dexamphetamine-induced locomotor hyperactivity in rats[21]. Taken together, these data suggest that 5-$HT_{2C}$ receptors exert an inhibitory influence on DA neurotransmission within the mesolimbic pathway[9]. Interestingly, studies using local administration of 5-$HT_{2C}$ receptor modulators indicate that 5-$HT_{2C}$ receptors located at the terminal level, i.e., nucleus accumbens, also contribute to the inhibitory control of DA neurotransmission[29].

In the SN pars reticulata, the dual 5$HT_{2B/2C}$ receptor agonist RO60-0175 was found to exert an excitatory effect of the majority of presumed GABAergic neurons[15]. In the same study, RO60-0175 also increased GABA levels in the SN pars reticulata, an effect attributed to 5-$HT_{2C}$ receptor stimulation since it was prevented by the selective 5-$HT_{2C}$ antagonist SB-243213. In another study, mCPP was reported to increase the firing rate of presumed GABAergic interneurons in both the SN pars reticulata and VTA via activation of 5-$HT_{2C}$ receptors, as indicated by the complete prevention of the effect of mCPP by SB-242084[16]. Interestingly, mCPP was found to affect GABAergic interneurons, but not projection neurons in the SN pars reticulata, while all non-DA neurons in the VTA were equally excited by mCPP. These findings may explain the differential response to mCPP and other 5-$HT_{2C}$ agonists, preferentially inhibiting the mesolimbic compared to the nigrostriatal dopaminergic function[16]. However, this preferential modulation of mesolimbic DA transmission by 5-$HT_{2C}$ receptors has been questioned based on findings indicating that 5-$HT_{2C}$ receptor stimulation or blockade alters DA levels to the same extent in the striatum and nucleus accumbens[30].

### 5-$HT_{2C}$ RECEPTOR MODULATION FOR THE TREATMENT OF SCHIZOPHRENIA

Schizophrenia is a psychiatric disorder affecting 1% of the population worldwide. Symptoms associated with schizophrenia have traditionally been categorized into positive symptoms (delusions and hallucinations), negative symptoms (blunting of affect, social withdrawal, lack of motivation), cognitive deficits (impairment in memory, executive function, working and long-term memory), and affective (depressive) symptoms[31,32]. The DA hypothesis of schizophrenia, implying a hyperactivity of the DA system, has been the main neurochemical hypothesis for many years. Current antipsychotic drugs offer some improvement of positive symptoms, but a very limited benefit on negative and cognitive symptoms. Furthermore, severe side effects, including extrapyramidal motor symptoms and weight gain, are often induced by current antipsychotic drugs. Therefore, the discovery of novel drugs with an improved side effect profile has been a major focus over the past years in schizophrenia research.

#### Antipsychotic Potential of 5-$HT_{2C}$ Receptor Modulation

5-$HT_{2C}$ receptor agonists have been proposed to offer potentially antipsychotic efficacy without traditional side effects associated with current antipsychotic drugs[33]. Interestingly, two selective 5-$HT_{2C}$ receptor agonists, WAY-163909 and CP-809101, were reported to exert antipsychotic-like effects in a dexamphetamine-induced locomotor hyperactivity paradigm in rats[34,35]. As expected, the 5-$HT_{2C}$ receptor antagonist SB-242084 was found to induce an opposite effect, i.e., enhanced dexamphetamine-induced locomotor hyperactivity[21], further supporting an inhibitory influence of 5-$HT_{2C}$ receptors on DA-induced behaviors. In addition, WAY-163909 produced a dose-dependent decrease in conditioned avoidance response behavior in rats[34], a model predictive of antipsychotic activity highly dependent on mesocorticolimbic DA transmission[36,37,38]. Similarly, the 5-$HT_{2C}$ receptor agonist CP-809101 has been shown to suppress the avoidance response dose dependently, supposedly through the 5-$HT_{2C}$

receptor, since a 5-HT$_{2A}$ receptor agonist did not show any effect on conditioned avoidance response behavior[35]. Taken together, these preclinical findings support an antipsychotic potential of 5-HT$_{2C}$ receptor agonists.

Controversy exists as to whether 5-HT$_{2C}$ receptors exert both a tonic and phasic, or only a phasic inhibitory influence on DA function[15,27]. For instance, the nonselective 5-HT$_{2C}$ receptor agonists mCPP and RO60-0175 were reported to decrease spontaneous locomotor activity in rats, an effect which could be reversed by the 5-HT$_{2C}$ receptor antagonists RO60-0491 and SB-242084[39,40]. In contrast, WAY-163909 has been shown to be devoid of effect on spontaneous locomotor activity in mice[34], which is consistent with an atypical antipsychotic profile of WAY-163909[41]. Electrophysiologically, WAY-163909 has been shown to selectively decrease the number of spontaneously active DA neurons in the VTA, but not in the SN pars compacta[34]. This selective effect in the VTA may be due to a differential activation of the 5-HT$_{2C}$ receptors on GABAergic interneurons in the VTA compared to the SN, as also suggested by others[16], and may also be responsible for the reported preferential reduction of DA levels in the nucleus accumbens[34]. As an alternative explanation, differences in 5-HT$_{2C}$ receptor isoforms in the nigrostriatal vs. mesolimbic system may also contribute to the preferential action of certain agonists on the mesolimbic pathway[34].

A controversial delayed onset of antipsychotic effect has been described for current antipsychotic drugs. It has been hypothesized that full depolarization blockade of DA neurons in the VTA would be required to obtain antipsychotic effect, which is only achieved after repeated administration of antipsychotic drugs[42]. Interestingly, both acute and chronic administration of WAY-163909 was found to decrease the number of spontaneously active DA neurons in the VTA in rats[34]. Even though the underlying mechanism was not investigated, an inhibitory modulation rather than a depolarization blockade of DA neurons is likely to be involved in the observed effects. In addition, the purported desensitization of 5-HT$_{2C}$ receptors after repeated stimulation[24] was further ruled out, since a sustained effect of WAY-163909 was observed after repeated administration in these preclinical studies[34].

### Cognitive-Enhancing Potential of 5-HT$_{2C}$ Receptor Modulation

Some recent studies indicate that 5-HT$_{2C}$ receptors might be involved in cognitive function and their modulation may thus add further benefit for the treatment of cognitive symptoms in schizophrenia. For instance, WAY-163909 has been shown to elevate both acetylcholine and DA levels in the prefrontal cortex of rodents[34]. Since a facilitation of cholinergic and DA neurotransmission in the prefrontal cortex has been hypothesized to be responsible for the cognitive-enhancing effects of some antipsychotic drugs[43], it has been proposed that WAY-163909 may also provide improvement in cognitive function[34]. In line with this assumption, another 5-HT$_{2C}$ agonist, CP-809101, was reported to improve cognitive performances of rodents in the novel object recognition test[35]. As an alternative neurochemical substrate for DA neurotransmission, the cortical glutamatergic system has been reported to be regulated by 5-HT$_{2C}$ receptors and suggested to play a key role in cognitive processes[44]. In fact, the dual 5-HT$_{2B/2C}$ agonist RO60-0175 was found to reverse the impairment in accuracy induced by a NMDA receptor antagonist in the 5-choice serial reaction time task[44]. Yet, other studies have reported contrasting findings. For instance, the 5-HT$_{2C}$ receptor antagonist SB-243213 was reported to improve reversal learning deficits in an animal model of schizophrenia[45]. SB-242084 was also found to promote spatial reversal learning in rats, an effect attributed to 5-HT$_{2C}$ receptors located in the orbitofrontal cortex[46]. These cognitive-enhancing effects of 5-HT$_{2C}$ receptor antagonism were suggested to be dependent, at least in part, on the facilitation of DA transmission. The selective 5-HT$_{2C}$ receptor antagonist SB-242084 was indeed reported to increase basal DA levels in the prefrontal cortex[47]. In addition, the dual 5-HT$_{2B/2C}$ receptor agonist RO60-0175 was shown to either not affect or suppress basal DA levels in the prefrontal cortex, and to inhibit stress-induced increase in DA release[47,48], effects further attributed to stimulation of 5-HT$_{2C}$ receptors. These findings are in agreement with the large body of evidence supporting an inhibitory role of 5-HT$_{2C}$ receptors on DA neurons in the midbrain. It is important to note that SB-242084 was also reported to impair performances in the 5-choice serial reaction time task[49,50]. In these studies, 5-HT$_{2C}$ receptor blockade was found to enhance premature responding consistently, a recognized measure of impulsivity. However, variable effects were observed on accuracy, a parameter more dependent on attentional processes. Overall, a potential benefit of 5-HT$_{2C}$ receptor modulation for the treatment of cognitive deficits associated with schizophrenia remains a matter of debate. Some preclinical findings support cognitive-enhancing properties of 5-HT$_{2C}$ receptor antagonism. However, other studies favor 5-HT$_{2c}$ receptor agonism, rather than antagonism, as potentially beneficial for cognitive function. One important consideration is the fact that the preclinical findings available rely on the use of different behavioral tasks assessing different cognitive domains, i.e., working memory, episodic memory, or attention, which may contribute to the existing disagreement related to the role of 5-HT$_{2C}$ receptor in the modulation of cognitive function.

In summary, 5-HT$_{2C}$ receptor modulation may represent an interesting target for the treatment of symptoms associated with schizophrenia. In line with this assumption, 5-HT$_{2C}$ receptor agonists have shown promising effects in several animal paradigms predictive of antipsychotic-like activity, and may also offer a more favorable side effect profile with respect to extrapyramidal symptom liability. Yet, whether 5-HT$_{2C}$ receptor antagonism or agonism may be beneficial for the treatment of cognitive symptoms associated with schizophrenia is still a subject of controversy. Vabicaserin, a 5-HT$_{2C}$ receptor agonist, is currently in development for the treatment of schizophrenia. The outcome of these clinical studies will be met with great interest and will hopefully reveal whether 5-HT$_{2C}$ receptors represent a viable target for the treatment of schizophrenia.

### 5-HT$_{2C}$ RECEPTOR MODULATION FOR THE TREATMENT OF MOOD DISORDERS

Major depression and anxiety disorders are severe disabling diseases that are highly prevalent and associated with negative impact on medical health, quality of life, and productivity[51,52]. Preclinical and clinical evidence suggest that depression is caused by a decreased availability of 5-HT and noradrenaline, since tricyclic antidepressants, e.g., imipramine, increase levels of both neurotransmitters in the cerebrospinal fluid through blockade of their respective transporter[53,54]. In contrast, anxiety is believed to result mainly from a hyperactive state of the 5-HT system and a dysfunction of 5-HT$_{1A}$ receptors has been suggested to play a key role[55]. The introduction of selective 5-HT reuptake inhibitors (SSRIs) and combined 5-HT and noradrenaline reuptake inhibitors into clinical practice has led to an improvement in the treatment of major depression and anxiety disorders by producing therapeutic benefit without the serious side effects associated with the older tricyclic antidepressants[56]. Although SSRIs and combined 5-HT and noradrenaline reuptake inhibitors are effective, a meaningful therapeutic improvement is only apparent after several weeks of treatment[57]. Furthermore, many depressed patients respond only partially and a substantial proportion of patients fail to respond at all to first-line treatment[58]. Moreover, in those patients that do respond, side effects, such as sexual dysfunction, sleep disturbances, and gastrointestinal disturbances, have been reported[59].

During the past years, various data support the idea that compounds blocking 5-HT$_{2C}$ receptors might be beneficial for the treatment of major depression and anxiety. For instance, the progressive therapeutic improvement achieved with classical antidepressant drugs is accompanied by a down-regulation of 5-HT$_{2C}$ receptor function[60,61], an observation that has led to the assumption that blockade of this receptor may offer therapeutic benefit. In addition, a large body of evidence indicates that stimulation of 5-HT$_{2C}$ receptors induces anxiogenic responses in both humans and rodents[62,63,64], further supporting a potential benefit of 5-HT$_{2C}$ antagonists as anxiolytic drugs. In contrast, few reports seem to indicate that 5-HT$_{2C}$ receptor agonists, rather than antagonists, may offer antidepressant benefit[65,66,67]. The most compelling evidence from the literature and the underlying mechanisms are reviewed in the following sections.

### Antidepressant Potential of 5-HT$_{2C}$ Receptor Modulation

Several lines of evidence suggest that antagonizing 5-HT$_{2C}$ receptors may be relevant for the treatment of major depression. Altered patterns of 5-HT$_{2C}$ receptor editing were detected in postmortem brains from suicide victims with a history of major depression[22]. Preclinically, a depressive phenotype can be induced in animals by repeated inescapable shocks. This model, known as the learned helplessness model, was associated with alterations in RNA editing of the gene encoding 5-HT$_{2C}$ receptors[23]. A hyperfunctionality of 5-HT$_{2C}$ receptors has also been reported in Flinders Sensitive Line rats, another animal model of depression[68]. Interestingly, chronic treatment with antidepressants was reported to desensitize 5-HT$_{2C}$ receptors in normal rats[69] and restore 5-HT$_{2C}$ receptor function in Flinders Sensitive Line rats[68].

5-HT$_{2C}$ receptors are expressed throughout the corticolimbic system. A large body of evidence exists supporting an inhibitory role of 5-HT$_{2C}$ receptors on DA function[70]. Restoration of DA function has been proposed as a novel approach for the development of antidepressant therapies[71], since decreased DA transmission is believed to be associated with anhedonia, one of the major symptoms of depression[72,73]. In agreement with this hypothesis, SSRIs inhibit DA firing activity in the VTA, an effect attributed to stimulation of 5-HT$_{2C}$ receptors[74]. This inhibition of DA function by SSRIs has been suggested to hamper their antidepressant effects and 5-HT$_{2C}$ antagonists have therefore been suggested to represent potential effective adjuncts in SSRI-resistant depression[74]. In agreement with this assumption, the selective 5-HT$_{2C}$ antagonist SB-242084 was found to restore the antidepressant-like effect of citalopram, an SSRI, in DBA/2N mice that do not respond to SSRIs alone[75]. In addition, Flinders Sensitive Line rats were reported to show both decreased DA levels and increased 5-HT$_{2C}$ receptor function in the nucleus accumbens, which were restored by repeated antidepressant treatment[68].

In addition to a potential role of DA transmission in the antidepressant effect of 5-HT$_{2C}$ receptor blockade, several reports have shown that 5-HT$_{2C}$ receptor antagonists potentiated the effect of SSRIs on 5-HT transmission[76,77,78]. This is particularly important in view of the assumption that the delayed onset of antidepressant effect classically observed with SSRIs would result from an acute inhibitory effect on 5-HT cell firing via activation of 5-HT$_{1A}$ autoreceptors[79,80], leading in turn to an attenuated release of 5-HT at the terminal level. It has therefore been suggested that a faster onset of effect would be achieved by preventing the initial inhibitory effect of SSRIs at the somatodendritic level, leading to an overall increased 5-HT neurotransmission. Interestingly, SB-242084 was found to prevent the inhibitory effect of citalopram on 5-HT cell firing in the dorsal raphe nucleus[81], which is likely to contribute to the reported potentiation of citalopram-induced 5-HT release by SB-242084 in both the hippocampus and frontal cortex[76,77,78,81]. This potentiation was further shown to be dependent on 5-HT$_{2C}$ receptors located at the terminal level rather than in the dorsal raphe nucleus[81]. Taken together, these findings indicate that 5-HT$_{2C}$ receptor blockade may prove an effective way to augment the therapeutic effect of SSRIs.

In addition to a potential benefit of 5-HT$_{2C}$ receptor blockade as an adjunct therapy to available antidepressants, few studies have suggested that 5-HT$_{2C}$ receptor antagonists alone may also exhibit antidepressant properties. For instance, the potent 5-HT$_{2C}$ receptor antagonist S-32006 was recently reported to exert antidepressant-like effects in rodents, which were attributed to increased levels of DA and noradrenaline in the frontal cortex[82]. In addition, agomelatine, a melatonin agonist with 5-HT$_{2C}$ receptor antagonism properties, has shown promising antidepressant effects in clinical studies[83]. Interestingly, agomelatine was reported to increase frontocortical DA and adrenergic transmission in rats[84], an effect attributed to its 5-HT$_{2B/2C}$ receptor antagonism rather than melatonin receptor agonism properties, and which was further hypothesized to underlie its antidepressant effects. Even though the specific involvement of 5-HT$_{2C}$ receptors was not investigated in the latter study, an involvement of 5-HT$_{2B}$ receptors in the effects of agomelatine was ruled out, based on previous findings by the same authors[85]. However, at present, no clinical evidence supports that selective blockade of 5-HT$_{2C}$ receptors alone is sufficient to produce antidepressant effects in humans.

In contrast, selective activation of 5-$HT_{2C}$ receptors has also been suggested to offer potentially antidepressant benefit. For instance, WAY-161503 has been reported to exhibit antidepressant-like effects in rodents as assessed by the forced swimming test[65]. In the same studies, SB-206533, a mixed 5-$HT_{2B/2C}$ receptor antagonist, was found to block the antidepressant-like effects of both WAY-161503 and SSRIs, further supporting a potential benefit of 5-$HT_{2C}$ receptor agonism for the treatment of depression. This apparent inconsistency between the antidepressant properties of 5-$HT_{2C}$ receptor antagonist vs. agonist may well be explained by the fact that repeated stimulation of 5-$HT_{2C}$ receptors rapidly induces desensitization both *in vitro*[86,87] and *in vivo*[88], ultimately leading to a decrease in 5-$HT_{2C}$ receptor-mediated transmission. Interestingly, a down-regulation of 5-$HT_{2C}$ receptors has been reported after chronic treatment with SSRIs and suggested to contribute to their therapeutic effect[69]. As an alternative explanation, activation of 5-$HT_{2C}$ receptors has been reported to induce neurogenesis in the hippocampus[89], which may contribute to a potential antidepressant effect.

In summary, there is compelling evidence supporting an antidepressant potential of 5-$HT_{2C}$ receptor antagonism, either alone or as adjunct therapy. In addition, 5-$HT_{2C}$ receptor agonism has been shown to exert antidepressant-like effects in preclinical models. Whether the effect of agonists involve a desensitization of the receptor or a different population of 5-$HT_{2C}$ receptors[90] has not been clearly addressed yet.

#### Anxiolytic Potential of 5-$HT_{2C}$ Receptor Modulation

A large body of evidence supports the anxiolytic potential of 5-$HT_{2C}$ receptor antagonism. For instance, the selective 5-$HT_{2C}$ antagonist SB-242084 reduced anxiety-like behavior in rats, as assessed by the social interaction test[91]. In addition, SB-242084 blocked anxiogenic-like responses induced by ethanol withdrawal[92] as well as those induced by SSRIs or a nonselective 5-$HT_{2C}$ receptor agonist mCPP[91]. The novel 5-$HT_{2C}$ receptor antagonist S-32006 showed anxiolytic properties in Vogel conflict and social interaction tests in rats[82]. In addition to these preclinical findings, a recent clinical study showed that agomelatine was effective in the treatment of generalized anxiety disorder[93]. This anxiolytic effect of agomelatine was attributed to 5-$HT_{2C}$ receptor blockade in preclinical studies[94].

Studies aimed at investigating the neural substrate underlying the modulation of anxiety behaviors by 5-$HT_{2C}$ receptors suggest that different populations of 5-$HT_{2C}$ receptors may exert different roles. For instance, mCPP application in the periaqueductal gray was found to induce anxiolytic effect in the elevated plus maze in rats via activation of 5-$HT_{2C}$ receptors[95], while it was found to induce anxiogenic responses when administered systemically[91]. Interestingly, 5-$HT_{2C}$ receptor knockout mice exhibit an anxiolytic-like phenotype, which was further attributed to a blunted activation of the extended amygdala by anxiogenic stimuli[96].

In summary, it appears that 5-$HT_{2C}$ receptor blockade may hold promise as a putative novel anxiolytic drugs.

### 5-$HT_{2C}$ RECEPTOR MODULATION FOR THE TREATMENT OF OBESITY

Overweight and obesity, defined as having a body mass index equal to or more than 25 and 30 $kg/m^2$, respectively, are major global health issues affecting an estimated 2 billion adults in 2005, with an estimated rise to 3 billion in 2015. Overweight and obesity are major risk factors for chronic diseases, such as type II diabetes, cardiovascular diseases, including coronary heart disease, hypertension, and stroke, and some cancers[97].

The serotonergic system has long been known to be involved in feeding and energy balance, and antiobesity drugs such as sibutramine and dexfenfluramine act on the serotonergic system by inhibiting reuptake and release of 5-HT[98]. Both of them have considerable side effects, including increased risk of cardiovascular and cardiopulmonary diseases, which have led to the withdrawal of both compounds from the EU market and dexfenfluramine from the North American market[98,99]. Especially, activity at the 5-$HT_{2A}$ and 5-$HT_{2B}$ receptor has been associated with these side effects, since agonism at the 5-$HT_{2A}$ and 5-$HT_{2B}$ receptor has been associated with hallucinogenesis[4] and valvular hypertrophy, respectively[100,101].

Substantial evidence supports that the 5-$HT_{2C}$ receptor is critical for the anorectic effect of serotonergic activation and the satiating effects of dexfenfluramine has thus been demonstrated to be attenuated in 5-$HT_{2C}$ receptor knockout mice, as well as by selective 5-$HT_{2C}$ receptor antagonists[102,103,104].

The phenotype of 5-$HT_{2C}$ receptor knockout mice is characterized by hyperphagia, depressed metabolic rate, and disruption in satiety, which results in midlife obesity (5–6 months of age) accompanied by hyperinsulinemia and hyperleptinemia[105,106]. Furthermore, these mice develop type II diabetes when placed on a high-fat diet[107]. Recent data suggest that these knockout mice differentiate from wild-type mice already at weaning, before weight gain occurs, as indicated by an increased food intake and decreased metabolic rate in pups[106]. Pharmacological studies also support a role of 5-$HT_{2C}$ receptors in food intake and energy balance. For instance, the nonselective 5-$HT_{2C}$ receptor agonist mCPP was reported to induce hypophagia and weight loss in rats, which were attenuated by the 5-$HT_{2C}$ receptor antagonist SB-242084[39,108] and absent in 5-$HT_{2C}$ receptor knockout mice[105]. In humans, mCPP has been demonstrated to decrease food intake, presumably through reducing the subjective feeling of hunger[109], and causes a small, but significant, weight loss in obese patients[110]. However, these effects were accompanied by transient increases in blood pressure and heart rate, as well as nausea, anxiety, and lightheadedness[109,110]. The more selective 5-$HT_{2C}$ receptor agonist WAY-163909 was further shown to reduce food intake and body weight in both lean and obese rodents[111], although at doses higher than those showing antipsychotic-like effects[34]. In the same study, the hypophagia and weight loss induced by acute administration of WAY-163909 were persistent after chronic administration, further ruling out a possible tolerance to the effects of sustained 5-$HT_{2C}$ receptor stimulation, as also reported with various other 5-$HT_{2C}$ receptor agonists[112,113,114,115]. The regulation of energy homeostasis by 5-$HT_{2C}$ receptors has been suggested to involve the melanocortin system. In agreement, the anorectic effects of dexfenfluramine were reported to be absent in melanocortin 3 and 4 receptor knockout mice[116]. Recently, it was further shown that 5-$HT_{2C}$ receptors expressed on pro-opiomelanocortin neurons were sufficient to mediate the acute anorexigenic effects of serotonergic drugs[117].

Lorcaserin (APD-356) is a 5-$HT_{2C}$ receptor agonist[118] found to result in significant body weight reduction in obese patients in a phase III study, accompanied by decreased cardiovascular risk factors and improved quality of life. BVT-933 (now PRX-00933), another 5-$HT_{2C}$ receptor agonist, was reported to induce significant weight reduction in a phase II trial. It is noteworthy that clinical development of other 5-$HT_{2C}$ receptor agonists has been discontinued, although most likely for safety issues related to activity at other serotonergic receptors, including 5-$HT_{2A}$ and 5-$HT_{2B}$. In conclusion, the promising results with lorcaserin suggest that highly selective 5-$HT_{2C}$ receptor agonists may be central in the pharmacological treatment of obesity.

### CONCLUSIONS

Substantial evidence supports a therapeutic potential of 5-$HT_{2C}$ receptor modulation in the treatment of a variety of pathological conditions, including schizophrenia, mood disorders, and obesity (Table 2). While both preclinical and clinical evidence support the benefit of 5-$HT_{2C}$ receptor agonists in the treatment of obesity, equivocal findings exist in the literature with respect to the potential benefit for mood disorders. While 5-$HT_{2C}$ receptor antagonism has been proposed to provide potentially antidepressant and anxiolytic effects, few contrasting studies have suggested that 5-$HT_{2C}$ receptor agonists, rather than antagonists, may offer a therapeutic benefit. The reason for these paradoxical data is not clear, but some studies suggest that the existence of different populations of 5-$HT_{2C}$ receptors, i.e., different localization, isoforms, and/or

**TABLE 2**
**Main Preclinical Evidence Supporting a Therapeutic Potential for 5-$HT_{2C}$ Receptor Modulation**

| Tool | Effect | Ref. |
|---|---|---|
| **Schizophrenia** | | |
| WAY-163909 | ↓ Dexamphetamine-induced locomotor hyperactivity in rats | [34] |
| | ↓ Conditioned avoidance response in rats | |
| | ↓ Number of active DA neurons in VTA, but not in SN, after acute and chronic administration | |
| | ↓ Basal DA levels in nucleus accumbens | |
| | ↑ Basal DA and acetylcholine levels in prefrontal cortex | |
| CP-809101 | ↓ Dexamphetamine-induced locomotor hyperactivity in rats | [35] |
| | ↓ Conditioned avoidance response in rats | |
| | ↑ Perfomances in novel object recognition in rats | |
| RO60-175 | ↔ or ↓ Basal DA levels in prefrontal cortex | [44,47,48] |
| | ↓ Stress-induced DA release in prefrontal cortex | |
| | Reverses NMDA antagonist-induced impairment in 5-choice serial reaction time task | |
| SB-242084 | ↑ Dexamphetamine-induced locomotor hyperactivity in rats | [21,46,47] |
| | ↑ Basal DA levels in prefrontal cortex | |
| | ↑ Spatial reversal learning in rats | |
| SB-243213 | ↑ Reversal learning in animal model of schizophrenia | [45] |
| **Depression** | | |
| Flinders Sensitive Line rats | ↑ 5-HT2C receptor function and ↓ DA levels in nucleus accumbens | [68] |
| SB-242084 | ↑ Antidepressant-like effect of citalopram in SSRI-resistant DBA/2A mice | [68,75,77, 78,81] |
| | Reverses inhibitory effect of citalopram on DA neuronal activity | |
| | Prevents inhibitory effect of citalopram on 5-HT neuronal activity | |
| | ↑ Citalopram-induced 5-HT release in prefrontal cortex and hippocampus | |
| S-32006 | Acute antidepressant-like effects in forced swim test in rats | [82] |
| | Antidepressant effect after repeated administration in a chronic mild stress procedure | |
| | ↑ DA and noradrenaline levels in prefrontal cortex | |
| Agomelatine | ↑ DA and noradrenaline levels in prefrontal cortex | [84,124] |
| | Antidepressant-like effects in learned helplessness, forced swim and chronic mild stress models in rats | |
| WAY-161503 | Antidepressant-like effect in forced swim test in rats | [65] |
| **Anxiety** | | |
| 5-$HT_{2C}$ knockout mice | ↓ Anxiety-like behavior in elevated zero maze and open field tests | [96] |
| SB-242084 | ↓ Anxiety-like behavior in social interaction test rats | [91,92] |
| | ↓ Anxiogenic-like responses induced by ethanol withdrawal | |
| S-32006 | ↓ Anxiety-like behavior in Vogel conflict and social interaction tests rats | [82] |
| Agomelatine | ↓ Anxiety-like behavior in Vogel conflict, social interaction, and elevated plus-maze tests rats | [90] |
| mCPP | ↓ Anxiety-like behavior in elevated plus-maze in rats after intraperiaqueductal gray injection | [91,95] |
| | ↑ Anxiety-like behavior in social interaction test in rats after systemic administration | |
| **Obesity** | | |
| 5-$HT_{2C}$ knockout mice | ↑ Food intake, ↓ metabolic rate, altered statiety, midlife obesity | [105,106, 117] |
| | Hyperphagia and obesity reversed by re-expression of 5-$HT_{2C}$ receptor in hypothalamic pro-opiomelanocortin neurons | |
| mCPP | ↓ Food intake and body weight, absent in 5-$HT_{2C}$ knockout mice | [105,108] |
| WAY-163909 | ↓ Food intake and body weight in lean and obese rats | [111] |

↑: increase; ↓: decrease; ↔ no change.

signaling, may partly contribute to some of the discrepancies reported in the literature. Furthermore, 5-$HT_{2C}$ receptors have also been proposed as a target relevant in schizophrenia research, primarily due to the interaction with DA pathways. However, while 5-$HT_{2C}$ receptor agonism may provide antipsychotic efficacy, whether 5-$HT_{2C}$ antagonism or agonism may be beneficial for the treatment of cognitive deficits associated to schizophrenia remains debated. Overall, the outcome from clinical trials with selective compounds in anxiety, depression, and schizophrenia will be met with great interest and will hopefully reveal whether 5-$HT_{2C}$ receptors represent a viable target for the treatment of these disorders.
